# Platelet-to-lymphocyte ratio as a biomarker of systemic inflammation in systemic lupus erythematosus: A meta-analysis and systematic review

**DOI:** 10.1371/journal.pone.0303665

**Published:** 2024-05-16

**Authors:** Young Ho Lee, Gwan Gyu Song

**Affiliations:** Department of Rheumatology, Korea University College of Medicine, Seoul, Korea; Transilvania University of Brasov: Universitatea Transilvania din Brasov, ROMANIA

## Abstract

**Objective:**

The objective of this study was to evaluate the relationship between the platelet-to-lymphocyte ratio (PLR) and systemic lupus erythematosus (SLE). Additionally, the study aimed to establish an association between PLR and SLE disease activity, specifically lupus nephritis (LN).

**Methods:**

We conducted a comprehensive search across Medline, Embase, and Cochrane databases to identify relevant articles. Subsequently, we performed meta-analyses to compare PLR between SLE patients and controls, as well as active and inactive SLE cases, along with LN and non-LN groups. Furthermore, a meta-analysis was conducted on correlation coefficients between PLR and various parameters in SLE patients, including the SLE Disease Activity Index (SLEDAI), C3, C4, anti-dsDNA, erythrocyte sedimentation rate (ESR), and C-reactive protein (CRP).

**Results:**

In total, fifteen studies comprising 1,522 SLE patients and 1,424 controls were eligible for inclusion. The meta-analysis demonstrated a significant elevation of PLR in the SLE group compared to the control group (Standardized Mean Difference [SMD] = 0.604, 95% Confidence Interval [CI] = 0.299–0.909, p < 0.001). Upon stratification by ethnicity, an elevated PLR was observed in the SLE group among both Asian and Arab populations. Subgroup analysis based on sample size revealed consistently higher PLR in both small (n < 200) and large sample (n ≥ 200) SLE groups. Moreover, when considering disease activity, there was a noteworthy trend of increased PLR in the active disease group compared to the inactive group (SMD = 0.553, 95% CI = 0.000–1.106, p = 0.050). However, the meta-analysis did not demonstrate a significant distinction in PLR between the LN and non-LN groups. Notably, a positive association was established between PLR and SLEDAI (correlation coefficient = 0.325, 95% CI = 0.176–0.459, p < 0.001). Furthermore, PLR exhibited positive correlations with ESR, CRP, proteinuria, C3, and anti-dsDNA antibody levels.

**Conclusions:**

The outcomes of this meta-analysis underscored the elevated PLR in SLE patients, suggesting its potential as a biomarker for gauging systemic inflammation in SLE. Additionally, PLR exhibited correlations with SLEDAI, as well as with key indicators such as ESR, CRP, proteinuria, C3, and anti-dsDNA antibody levels.

## Introduction

Systemic Lupus Erythematosus (SLE) is a chronic autoimmune disorder characterized by a dysregulated immune response that leads to the production of autoantibodies and subsequent inflammation, affecting multiple organ systems [1]. The pathogenesis of SLE involves complex interactions among genetic predisposition, environmental triggers, and immune dysregulation, resulting in a wide range of clinical manifestations and disease severity [2]. Despite significant advances in understanding the underlying mechanisms of SLE, its etiology remains elusive, and the quest for reliable biomarkers for monitoring disease activity and predicting outcomes remains ongoing.

In recent years, there has been a growing interest in the role of hematological indices as potential indicators of systemic inflammation and disease severity in various inflammatory conditions, including SLE. Among these, platelet-to-lymphocyte ratio (PLR) has emerged as a promising candidate owing to its simplicity, cost-effectiveness, and potential association with immune-inflammatory processes [3]. The PLR reflects the balance between two essential components of the immune system: platelets, which are crucial in initiating and propagating inflammation, and lymphocytes, the primary mediators of immune responses [4]. Understanding the relationship between the PLR and SLE may have significant clinical implications. If PLR is a reliable indicator of disease activity, it could aid in risk stratification, guide treatment decisions, and monitor therapeutic responses. Additionally, identifying potential ethnic differences in PLR and their correlation with laboratory findings could enhance our understanding of the heterogeneity of the disease and potentially facilitate personalized management strategies for patients with SLE.

Several studies have investigated the relationship between the PLR and SLE; however, the findings are inconsistent and sometimes contradictory [5–19]. Some studies have reported elevated PLR in patients with SLE compared to healthy controls, whereas others have failed to show a significant difference. Moreover, the association between the PLR and disease activity or laboratory findings in patients with SLE remains incompletely understood. In light of these uncertainties, we conducted a comprehensive meta-analysis to evaluate the association between the PLR and SLE. By pooling data from multiple studies, this meta-analysis aimed to provide a more precise estimation of the relationship between the PLR and SLE, determine whether the PLR can serve as a potential biomarker for assessing disease activity and severity, and explore its correlation with commonly used laboratory parameters.

## Materials and methods

### Choosing appropriate studies and gathering data

We searched the literature for studies that examined the PLR in patients with SLE and healthy controls. The MEDLINE, Embase, and Cochrane databases (up to July 2023) were searched to identify all accessible papers. The search was conducted using the phrases "platelet to lymphocyte ratio" and "systemic lupus erythematosus" as keywords and topic terms. To identify other studies not included in the aforementioned electronic databases, all references listed in the identified publications were also examined. Studies were deemed eligible if they met one or more of the following criteria: (1) case-control, cross-sectional, or cohort studies; (2) studies providing information on the PLR in patients with SLE and controls; or (3) studies providing information on the correlation coefficient between the PLR and SLE activity as measured by the SLEDAI, ESR, and C3, C4, anti-dsDNA, or CRP levels. There were no language- or race-based limitations in this study. Studies were disregarded if they were reviews or case reports or if they had overlapping or inadequate data. Two independent reviewers collected data on the procedures and outcomes of the original trials. Any disagreements in the conclusions were resolved by consensus. The PRISMA recommendations were followed in conducting the meta-analysis [20]. The main author, publication year, country, participant count, mean and standard deviation (SD) of the PLR, and correlation coefficients between the PLR and disease activity were extracted. The mean and SD values were calculated using the previously published methods when the provided data were median, interquartile range, or ranges [21,22]. The quality of each component of the meta-analysis was scored using the Newcastle-Ottawa Scale [23].

### Examination of statistical relationships

We carried out a meta-analysis to ascertain the platelet-to-lymphocyte ratio (PLR) among patients with SLE in comparison to healthy controls, individuals with active and inactive SLE, and those with or without lupus nephritis (LN). The outcomes are expressed as standardized mean differences (SMDs) accompanied by 95% confidence intervals (CIs) to uphold data uniformity. Additionally, we conducted a meta-analysis examining the correlations between PLR and parameters such as SLEDAI, as well as levels of C3, C4, anti-dsDNA, ESR, and CRP within the SLE patient cohort. We assessed heterogeneity and variability within and across trials using Cochran’s Q test [24]. A heterogeneity test was used to investigate the null hypothesis that all the studies evaluated the same effect. A random-effects model was used in the meta-analysis when a substantial Q value (p < 0.10) showed study heterogeneity [25]. When a substantial Q statistic (p < 0.10) failed to detect study heterogeneity, the fixed-effects model was used. The model assumed that all studies assessed the same underlying effect and solely considered study heterogeneity. To determine the impact of heterogeneity, we used the following formula: *I*^2^ = 100% *×* (*Q* − *df*)/*Q* [26]. *I*^2^ was used to evaluate trial-to-trial consistency and determine whether heterogeneity, rather than chance, was primarily responsible for the majority of the total variation between studies. *I*^2^ levels of 25%, 50%, and 75% were considered low, moderate, and high, respectively. *I*^2^ ranged from 0% to 100% [26]. Statistical adjustments were made using Comprehensive Meta-Analysis computer software (Biostat, Inc., Englewood, NJ).

### Sensitivity test, heterogeneity assessment, and publication bias

Ethnicity, research quality, sample size, and data type were used as variables in meta-regression analyses to examine the probable origins of the heterogeneity observed in the meta-analysis. By excluding each study separately, a sensitivity test was conducted to determine the impact of each study on the pooled odds ratio. Although funnel plots are often used to identify publication bias, their interpretation requires judgment and various research types with different sample sizes. Therefore, we assessed publication bias using Egger’s linear regression test,[27] which was used to determine funnel plot asymmetry using a natural logarithm scale of SMDs.

## Results

### Studies included in the meta-analysis

**Fig 1:**
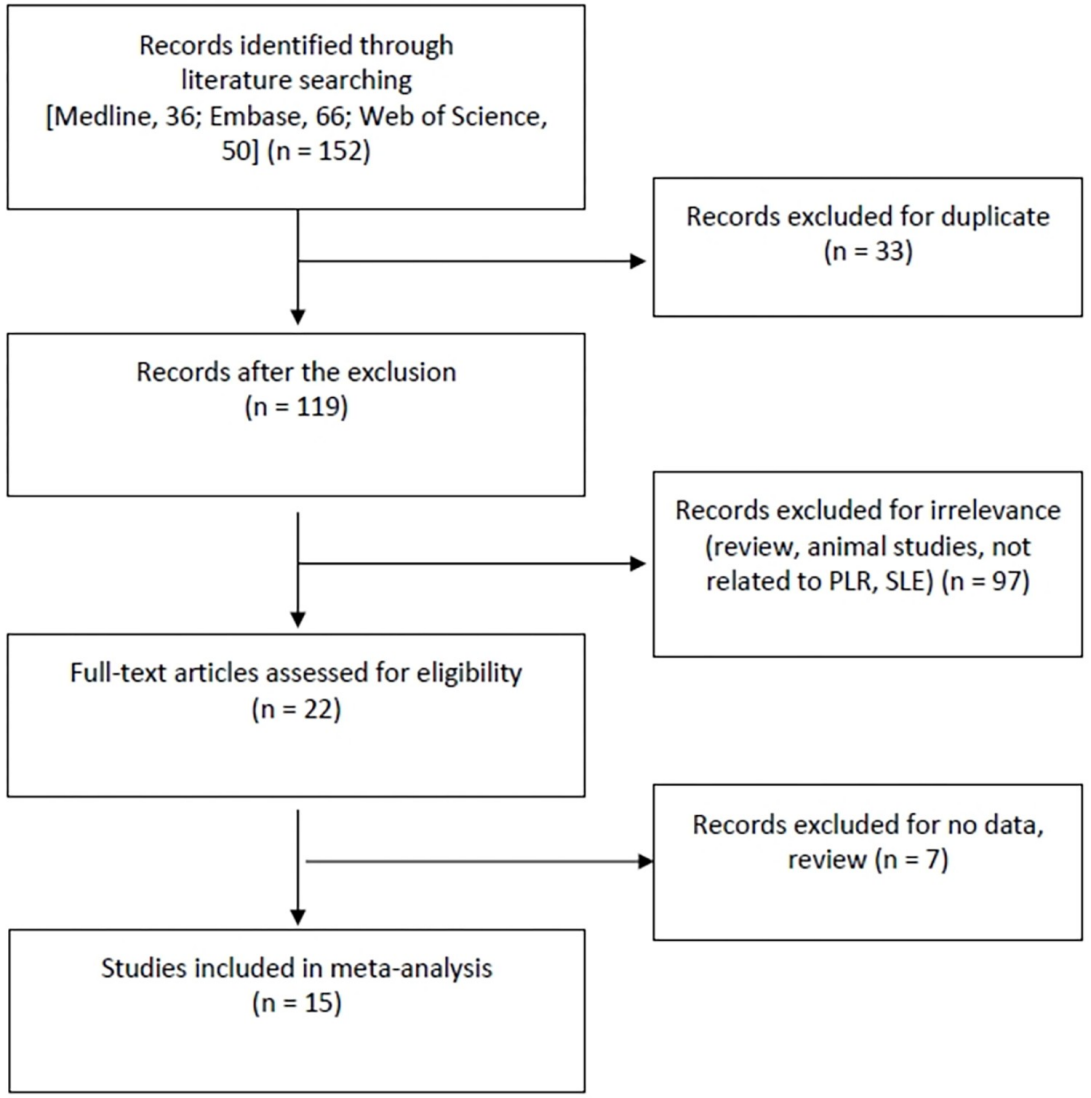
A diagram on choosing the relevant studies.

A comprehensive search utilizing both computerized and manual techniques initially yielded a total of 152 studies. Upon scrutiny of titles and abstracts, 21 papers were selected for in-depth analysis. Among these, seven were excluded due to either a lack of PLR data or their nature as review articles. Consequently, a total of 15 studies, encompassing 1,522 patients diagnosed with SLE and 1,424 control subjects, fulfilled the predefined inclusion criteria [5–19] (Table 1 and Fig 1). Each study was evaluated on a scale of 1 to 10, resulting in quality ratings ranging from 6 to 8. The distinctive attributes of the included studies are concisely summarized in Table 1.

**Table 1:** Characteristics of the individual studies included in the meta-analysis.

Authors	Country	Ethnicity	Groups	Number		SLEDAI, coefficient	Results			Study quality
				Case	Control		SMD*	Magnitude*	*p* Value	
**Abdalhadi, 2023[5]**	Syria	Arab	PLR	80	80	0.721	0.511	Medium	0.001	6
**Ozdemir, 2023[6]**	Turkey	European	PLR	76	76	0.214	-0.216	Small	0.183	6
**Moreno-Torres, 2022[7]**	Spain	European	PLR	77	80	0.183	1.202	Large	0.000	6
**Taha, 2022[8]**	Egypt	Arab	PLR	100	100	0.425	-0.590	Medium	0.000	7
**El-Said, 2022[18]**	Egypt	Arab	PLR	52	50	0.340	0.953	Large	0.000	6
**Abdulrahman, 2020[17]**	Egypt	Arab	PLR	110	50	0.640	2.309	Large	0.000	6
**Liu, 2020[9]**	China	Asian	PLR	56	57	-0.177	0.552	Medium	0.004	6
**Peirovy, 2020[10]**	Iran	Arab	PLR	208	205	0.340	1.180	Large	0.000	8
**Lao, 2020[11]**	China	Asian	PLR	195	183	0.312	0.465	Small	0.000	7
**Soliman, 2020[12]**	Egypt	Arab	PLR	120	30	NA	0.358	Small	0.081	6
**Gao, 2019[19]**	China	Asian	PLR	22	66	NA	-0.035	Small	0.885	6
**Xie, 2018[13]**	China	Asian	PLR	105	105	-0.159	0.666	Medium	0.000	7
**Wu, 2016[14]**	China	Asian	PLR	116	136	0.298	0.749	Medium	0.000	7
**Qin, 2016[15]**	China	Asian	PLR	154	151	0.440	0.461	Small	0.000	7
**Yolbas, 2016[16]**	Turkey	European	PLR	51	55	NA	0.533	Medium	0.007	6

SMD: Standardized mean difference, NLR: Neutrophil-to-lymphocyte ratio, PLR: Platelet-to-lymphocyte ratio, SLEDAI: Systemic lupus erythematosus disease activity Index, *Magnitude of Cohen’s *d* effect size: 0.2–0.5, small effect; 0.5–0.8, medium effect; ≥0.8, large effect; NA: Not available.

### Comparing PLR between SLE patients and controls

**Fig 2:**
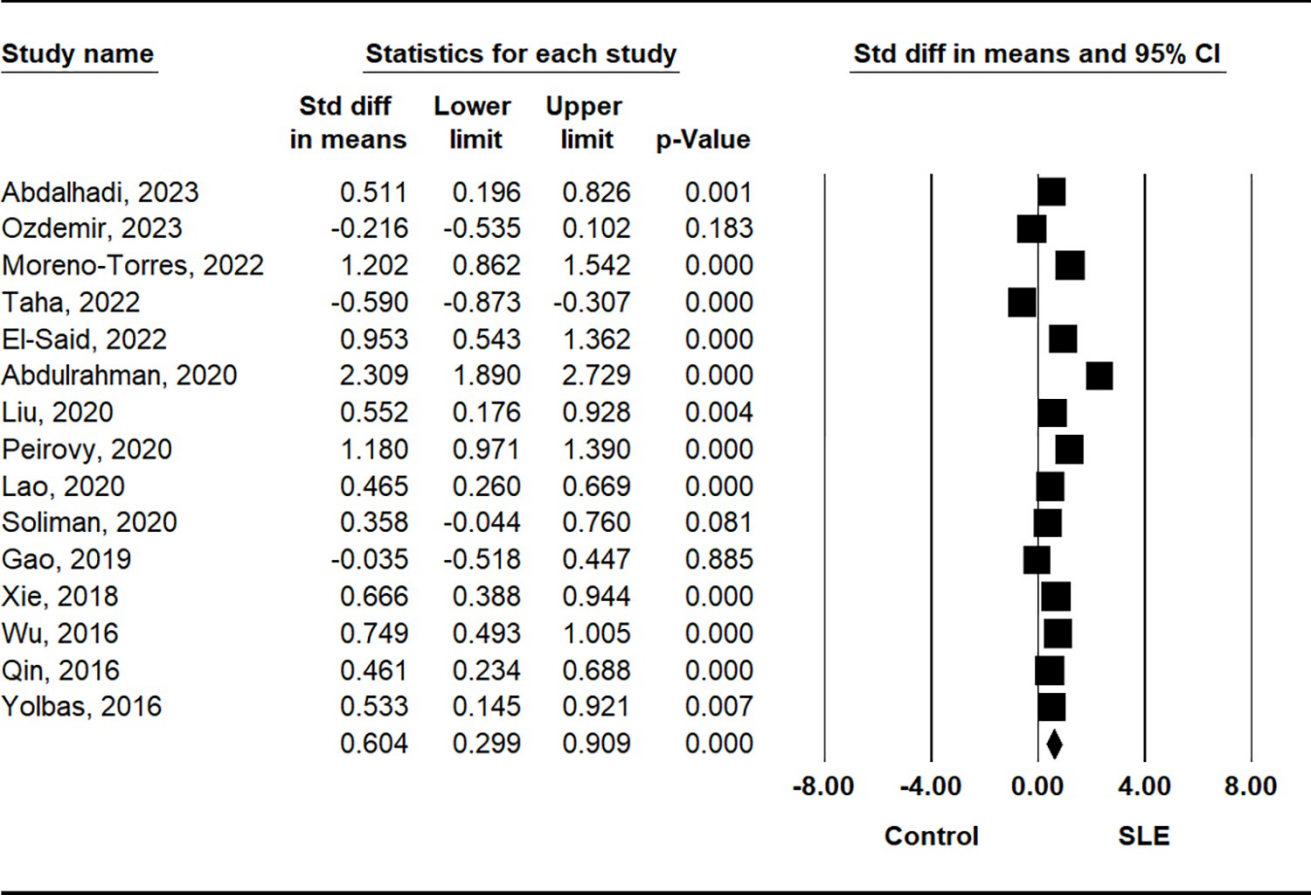
A meta-analysis of PLR in patients with SLE and controls.

**Fig 3:**
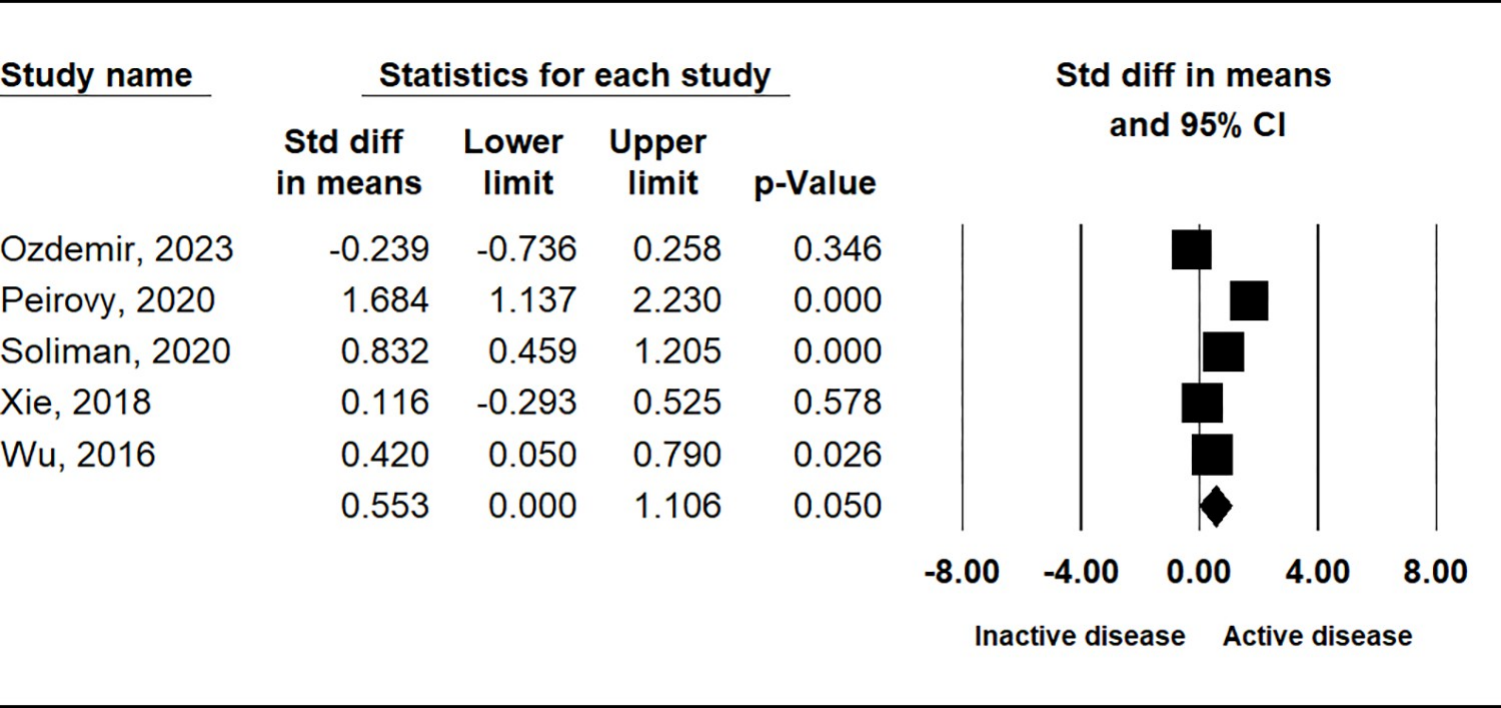
A meta-analysis of the correlation between PLR in groups with and without active disease.

**Fig 4:**
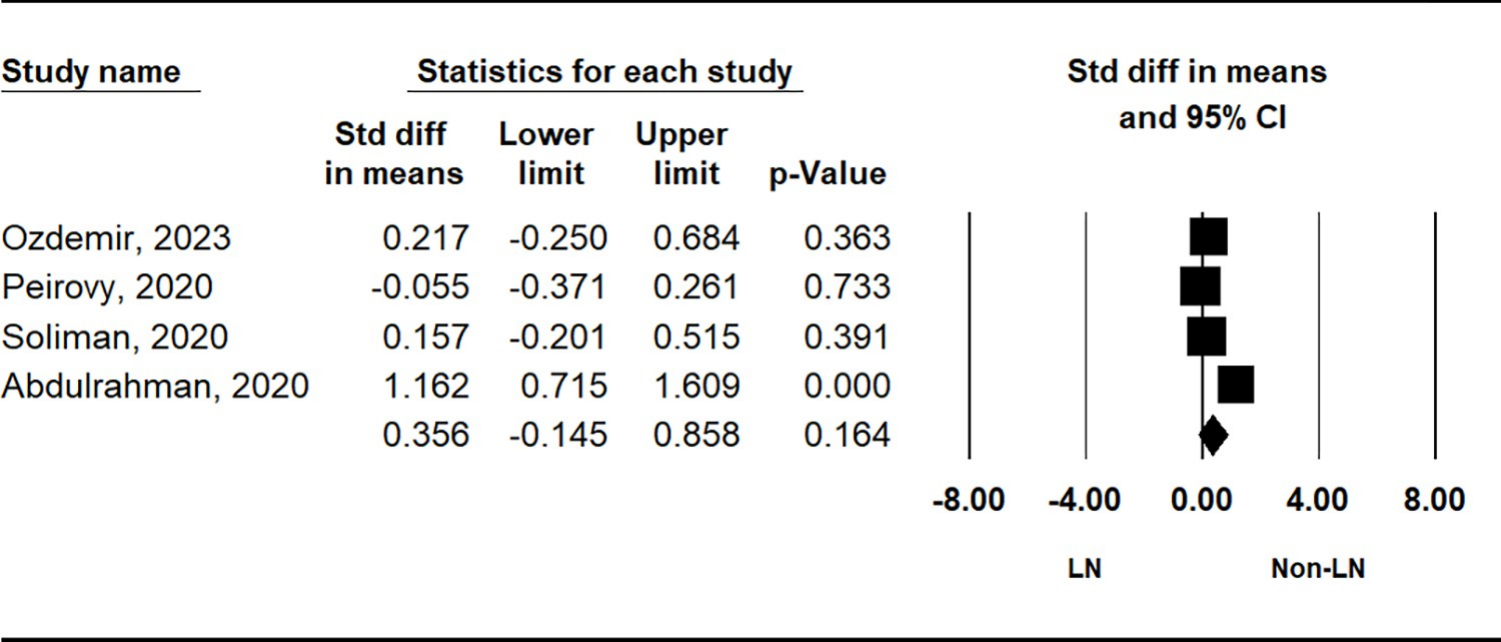
A meta-analysis of the correlation of PLR between LN and non-LN groups.

The comparison of PLR between patients with SLE and control subjects demonstrated a significant elevation in the SLE group (SMD = 0.604, 95% CI = 0.299–0.909, p = 0.001) (Table 2 and Fig 2). Stratification by ethnicity indicated notably higher PLR values in the SLE group among Asian and Arab populations, with no such trend observed in European populations (Table 2). Subgroup analysis based on sample size consistently revealed higher PLR in both small (n < 200) and large (n ≥ 200) sample size SLE groups (Table 2). Additionally, considering disease activity, the active disease group exhibited significantly higher PLR compared to the inactive disease group (SMD = 0.553, 95% CI = 0.000–1.106; p = 0.050) (Table 2 and Fig 3). However, no significant disparity in PLR was observed between the lupus nephritis (LN) and non-LN groups (Table 2 and Fig 4).

**Table 2:** Meta-analysis of PLR levels in SLE patients compared to that in controls.

Groups	Population	No. of studies	Test of association				Test of heterogeneity		
			SMD*	95% CI		*p*-value	Model	*p*-value	*I* ^2^
**All**	Overall	15	0.604	0.299–0.909	< 0.001		R	< 0.001	93.4
**Ethnicity**	Asian	6	0.520	0.359–0.681	< 0.001		R	0.082	48.7
	Arab	6	0.782	0.039–1.525	0.039		R	< 0.001	96.9
	European	3	0.505	0.344–1.353	0.244		R	< 0.001	94.2
**Sample size**	Small (< 200)	9	0.684	0.215–1.153	0.004		R	< 0.001	92.8
	Large (≥ 200)	6	0.492	0.053–0.932	0.028		R	< 0.001	95.0
**SLE activity**	Active vs. Inactive	5	0.553	0.000–1.106	0.050		R	< 0.001	87.9
**LN**	LN (+) vs. LN (-)	4	0.356	-0.145–0.858	0.164		R	< 0.001	84.8

PLR: Platelet to lymphocyte ratio, SLE: Systemic lupus erythematosus, LN: Lupus nephritis, CI: Confidence interval, F: Fixed effects model, R: Random effects model, NA: Not applicable.

*: Magnitude of Cohen’s d effect size (SMD): 0.2–0.5, small effect; 0.5–0.8, medium effect; ≥ 0.8, large effect.

### Correlation between PLR and clinical findings

**Fig 5:**
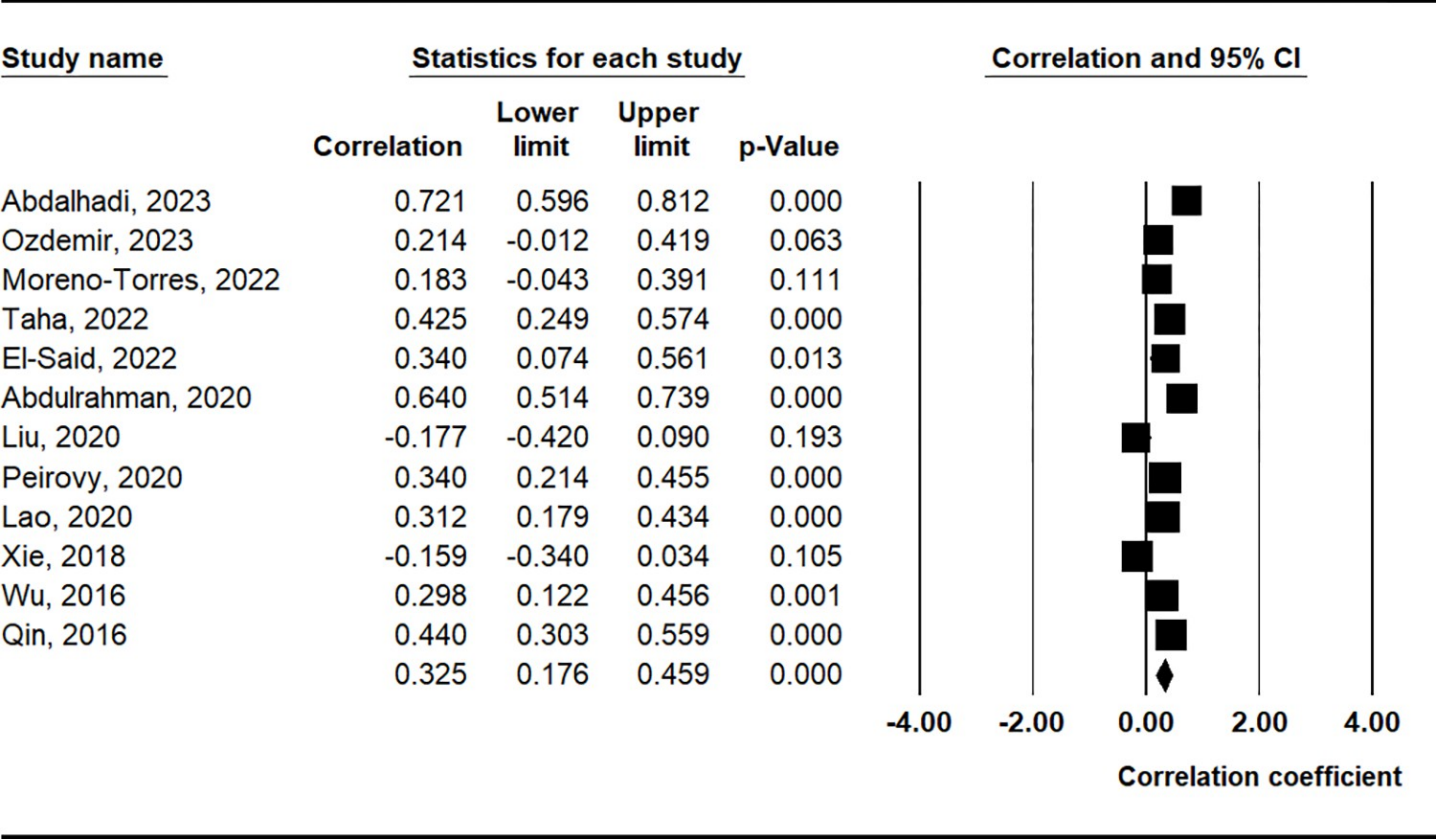
A meta-analysis of the correlation coefficient between the PLR and SLEDAI.

**Fig 6:**
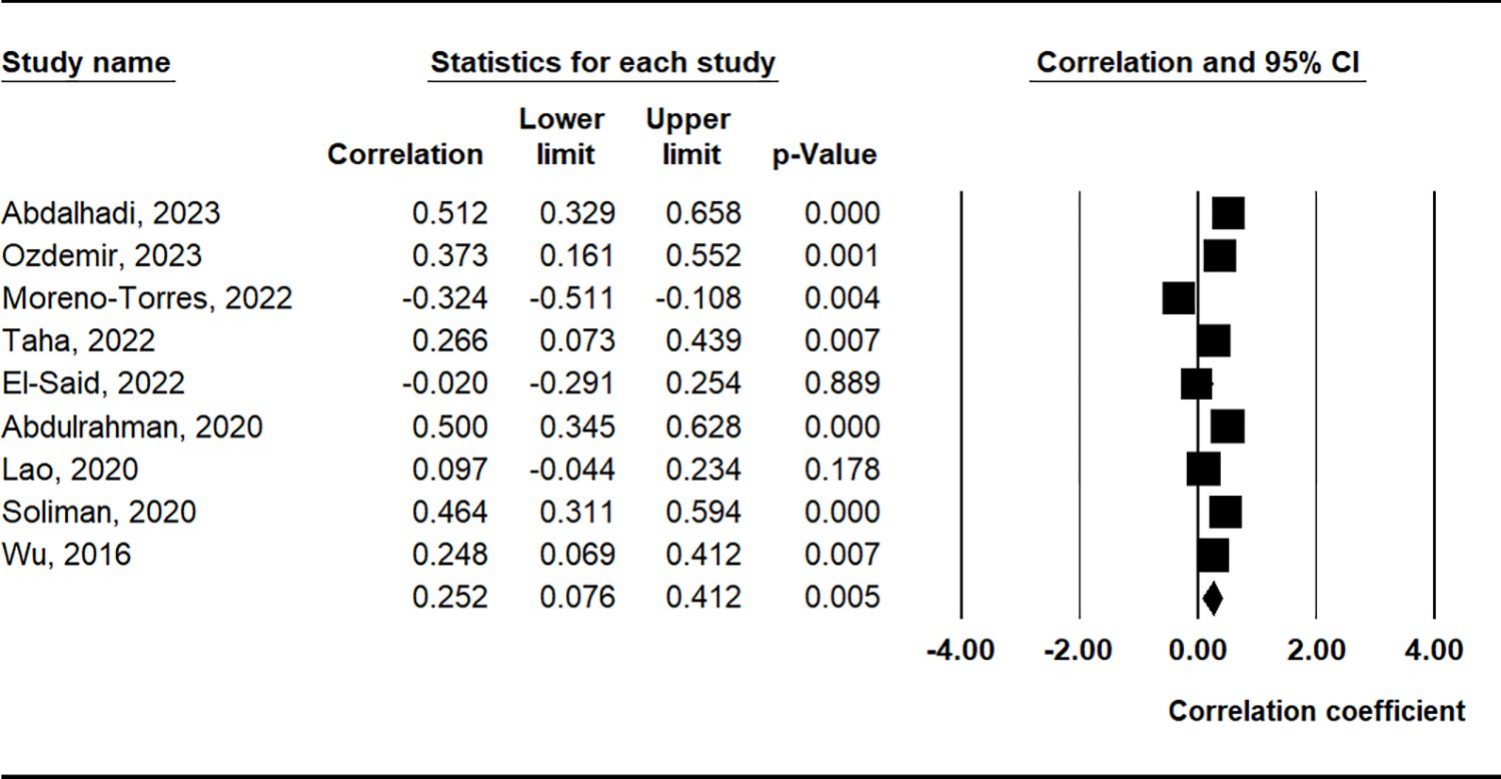
A meta-analysis of the correlation coefficient between the PLR and ESR.

The meta-analysis revealed a positive correlation between PLR and the SLEDAI (correlation coefficient = 0.325, 95% CI = 0.176–0.459, p < 0.001) (Table 3 and Fig 5). Furthermore, PLR demonstrated positive correlations with markers such as erythrocyte sedimentation rate (ESR), proteinuria, C-reactive protein (CRP), complement component C3, and anti-double-stranded DNA (anti-dsDNA) antibody levels (Table 3 and Fig 6).

**Table 3:** Meta-analysis of the correlation coefficient between PLR level and SLIDAI, C3, C4, ESR, CRP, proteinuria, and anti-dsDNA in SLE.

Parameters	No. of studies	Test of association			Test of heterogeneity		
		Correlation coefficient	95% CI	*p*-value	Model	*p*-value	I^*2*^
**SLEDAI**	12	0.325	0.176–0.459	< 0.001	R	< 0.001	87.8
**ESR**	9	0.252	0.076–0.412	0.005	R	< 0.001	86.5
**CRP**	8	0.215	0.119–0.307	< 0.001	R	0.040	52.2
**C3**	5	-0.280	-0.356- -0.200	< 0.001	F	0.405	0.260
**C4**	5	-0.181	-0.352–0.002	0.052	R	0.001	77.4
**Proteinuria**	4	0.298	0.088–0.483	0.006	R	0.005	75.8
**Anti-dsDNA**	1	0.325	0.137–0.490	0.001	NA	NA	NA

PLR: Platelet to lymphocyte ratio, SLEDAI: Systemic lupus erythematosus disease activity index, ESR: Erythrocyte sedimentation rate, CRP: C-reactive protein, CI: Confidence interval, R: Random effects model, NA: Not available.

### Sensitivity, heterogeneity, and publication bias

An assessment of PLR variations among SLE studies highlighted heterogeneity between studies (Tables 2 and 3). However, the major source of heterogeneity in the PLR meta-analysis stemmed from variations in the effect size. Notably, heterogeneity in the PLR meta-analysis was significantly influenced by data type (p = 0.001), whereas factors such as ethnicity, sample size, or research quality did not significantly impact it. The sensitivity analysis indicated that no individual study disproportionately affected the overall effect size, reinforcing the robustness of the meta-analysis findings. The funnel plot demonstrated symmetry, and the application of Egger’s regression test provided no indication of publication bias (p > 0.1).

## Discussion

The present meta-analysis showed significantly elevated PLR in the SLE group among the Asian and Arab populations but not among European populations, suggesting that there may be ethnicity-specific differences in the PLR-SLE relationship. However, it is essential to consider that the observed differences in the PLR among ethnic groups could be influenced by the number of studies available for each subgroup. As noted, there were only three European studies, compared to six studies in Asian and Arab populations. The limited number of European studies may have affected the precision and reliability of the estimates in this subgroup. Additionally, differences in study characteristics, patient demographics, and methodologies among the included studies may have contributed to the observed variations. Stratification of the PLR by ethnicity revealed further insights. Specifically, significantly elevated PLR was observed in the SLE group among Asian and Arab populations but not in the European populations. This finding suggests that ethnicity-specific differences may exist in the association between PLR and SLE. Genetic and environmental factors could contribute to these differences, warranting further investigation into the underlying mechanisms driving these disparities. Ethnicity-based variations in immune responses and inflammatory pathways may account for these differences, warranting further investigations into the underlying mechanisms driving these associations. Moreover, subgroup analysis based on sample size indicated that both small and large sample sizes in the SLE group showed significantly higher PLR than controls. This suggests consistency in the relationship between the PLR and SLE across different sample sizes, thereby enhancing the robustness of our findings. An important aspect of this study was the evaluation of the association between PLR and SLE disease activity. The results showed a trend of increased PLR in the active disease group compared with that in the inactive disease group, although the difference was not statistically significant. This trend suggests that the PLR may reflect disease activity to some extent; however, further research with larger cohorts is required to establish a definitive association.

We observed a positive association between PLR and SLEDAI, an established tool for assessing SLE disease activity. This correlation suggests that the PLR could serve as a complementary marker for disease activity assessment, particularly when SLEDAI scores are not readily available or feasible to obtain. Furthermore, the PLR was positively associated with ESR, proteinuria, and CRP, C3, and anti-dsDNA antibody levels. These findings indicate that the PLR may reflect not only systemic inflammation but also specific aspects of the pathogenesis of SLE, such as complement activation and renal involvement. These associations with laboratory parameters support the notion that PLR may reflect the overall inflammatory burden and disease severity in SLE. Interestingly, the meta-analysis did not find a significant difference in PLR between the LN and non-LN groups. Although this result may appear surprising, it underscores the complexity of SLE pathophysiology, as LN represents a distinct and severe manifestation of the disease. While PLR may exhibit a relationship with SLEDAI, this association does not necessarily imply a consistent link between LN and non-LN groups. SLE is a heterogeneous autoimmune disease with a wide array of clinical manifestations. LN represents a subset of patients with renal involvement, while others may predominantly exhibit extra-renal manifestations. The multifaceted nature of SLE implies that distinct immunopathogenic processes may be at play in different organ systems. PLR is generally associated with inflammatory processes. It is not specific to renal inflammation alone and may reflect systemic immune activation. Therefore, an association with SLEDAI may indicate an overall heightened immune response in SLE, irrespective of renal involvement. Future studies with larger LN cohorts could shed light on the role of the PLR in LN and its potential prognostic significance. There was a rationale for prioritizing the PLR marker above the neutrophil to lymphocyte ratio (NLR). The rationale for the choice was that a recent meta-analysis on the relationship between the NLR and Systemic Lupus Erythematosus (SLE) had previously been published [28].

Although this meta-analysis provides valuable insights into the association between the PLR and SLE, it had some limitations. First, the number of eligible studies was relatively small, which potentially limits the generalizability of the findings. Second, the included studies exhibited heterogeneity in terms of study design, patient characteristics, and PLR measurement methods, which may have influenced the results. Third, PLR is a non-specific marker for a number of diseases, including as autoimmune and inflammatory conditions. Since autoimmune disorders are heterogeneous, we concentrated on only SLE, a prototype of autoimmune disease, because its heterogeneity was low compared to autoimmune diseases. Nevertheless, one strength of this study was the rigorous and comprehensive nature of its meta-analysis. The study included a systematic literature search that identified nine relevant studies. This extensive data collection approach ensured that the findings were based on a large pool of data, thus enhancing the statistical power and generalizability of the results. By pooling data from multiple studies, the meta-analysis provides a more precise estimation of the relationship between the PLR and SLE. Meta-analyses are known for their ability to draw more robust conclusions than individual studies, especially when the effect size varies across different study populations and methodologies [29,30]. Thus, this meta-analysis strengthens the evidence of an association between the PLR and SLE, thereby increasing the credibility of the findings.

In conclusion, this meta-analysis demonstrated that PLR was higher in patients with SLE, with a significant positive correlation between PLR and SLEDAI, ESR, CRP, proteinuria, C3, and anti-dsDNA antibody levels. These findings support the potential of the PLR as a valuable biomarker for assessing systemic inflammation in SLE. However, further prospective studies with larger and more diverse cohorts are needed to validate these results and to determine the clinical utility of the PLR as a diagnostic and prognostic tool in SLE.

## Supporting information

S1 Checklist PRISMA 2020 checklist. (DOCX)
